# Progesterone and IL-6 Expression Are Modulated by Follicular Fluid in Granulosa Cell Cultures

**DOI:** 10.3390/biom15121646

**Published:** 2025-11-23

**Authors:** Loris Marin, Chiara Sabbadin, Claudia Maria Radu, Paola Brun, Carolina Frison, Giuseppe Gullo, Decio Armanini, Luciana Bordin, Eugenio Ragazzi, Guido Ambrosini, Alessandra Andrisani

**Affiliations:** 1 Department of Women’s and Children’s Health, University of Padova, 35128 Padova, Italy; loris.marin@unipd.it (L.M.); guido.ambrosini@unipd.it (G.A.); alessandra.andrisani@unipd.it (A.A.); 2 Endocrine Unit, Department of Medicine DIMED, University of Padova, 35128 Padova, Italy; chiara.sabbadin@unipd.it (C.S.); decio.armanini@unipd.it (D.A.); 3 Thrombotic and Haemorrhagic Diseases Unit, Department of Medicine DIMED, University of Padua, 35128 Padua, Italy; claudiamaria.radu@unipd.it; 4 Department of Molecular Medicine DMM, University of Padova, 35121 Padova, Italy; paola.brun.1@unipd.it (P.B.); carolina.frison@studenti.unipd.it (C.F.); 5 Department of Obstetrics and Gynecology, Villa Sofia Cervello Hospital, University of Palermo, 90146 Palermo, Italy; gullogiuseppe@libero.it; 6 Studium Patavinum, University of Padova, 35122 Padova, Italy; eugenio.ragazzi@unipd.it

**Keywords:** endometriosis, poor ovarian responder, granulosa cells, cortisol, estradiol, progesterone, IL-6, hormonal secretion, Il-6 expression

## Abstract

Endometriosis (ENDO) and poor ovarian response (POR) represent challenging conditions in assisted reproduction. Both, associated with altered follicular fluid (FF) composition, specifically impact on granulosa cell (GC) function in an incompletely understood way. GCs from male factor (MF, *n* = 30), ENDO (*n* = 38), and POR (*n* = 27) patients were cultured in media supplemented with FF from each group (FF-MF, FF-ENDO, FF-POR). Proliferation, morphology, and secretory activity (cortisol, estradiol, progesterone, IL-6) were assessed. GC proliferation depended primarily on FF origin, being highest with FF-ENDO, intermediate with FF-POR, and lowest with FF-MF. Morphological analysis revealed enrichment of muscle-like and fibroblast-like morphologies under FF-ENDO and FF-POR, suggestive of dysregulated luteinization and extracellular matrix remodeling. Secretory activity reflected a complex interplay between GC origin and FF type: IL-6 was strongly induced by FF-MF and FF-POR but consistently suppressed by FF-ENDO; cortisol and estradiol were generally consumed, while progesterone synthesis was largely confined to MF-GCs, with only variable induction in ENDO-GCs exposed to FF-POR. These findings indicate that pathological FF milieus reprogram GC behavior in distinct ways, with potential consequences for luteal function and oocyte competence. Identifying the molecular mediators of these alterations may guide tailored strategies to improve ART outcomes in ENDO and POR patients.

## 1. Introduction

Assisted reproductive technologies (ART), and in particular in vitro fertilization (IVF), have become essential tools for infertile couples. The number of women seeking IVF continues to grow globally, reflecting demographic shifts, delayed childbearing, and expanded access to reproductive medicine. Among women undergoing IVF, two patient subgroups present particular challenges: those affected by endometriosis (ENDO) and those classified as poor ovarian responders (POR), typically corresponding to Group 3 of the POSEIDON classification (younger women with a diminished ovarian reserve or reduced ovarian responsiveness [1,2]).

ENDO is a chronic estrogen-dependent, inflammatory disease characterized by ectopic growth of endometrial-like tissue outside the uterine cavity. It affects approximately 10% or more of women of reproductive age, and studies suggest that 25–50% of infertile women may harbor endometriosis. Through mechanisms involving chronic inflammation [3], oxidative stress [4,5,6], altered cytokine signaling [7] and local hormonal imbalances [8], endometriosis is widely considered to impair ovarian, tubal, and endometrial functions, thereby hindering fertility [9].

Poor ovarian response (POR)—and more broadly “low prognosis” in ART—describes patients who yield few oocytes despite standard stimulation protocols. The POSEIDON classification refined the concept by integrating age, ovarian reserve markers (AMH, AFC), and actual oocyte yield into four prognostic groups; Group 3 represents younger women (<35 y) with diminished reserve or hypo-responsiveness (AFC < ~5, AMH < ~1.2 ng/mL) [1]. These patients pose a paradox: although young, their ovarian response is poor, and conventional strategies (higher FSH, LH supplementation) often fail to rescue outcome [10].

Among the molecular changes implicated in endometriosis-associated follicular dysfunction, IL-6 and cortisol have drawn attention. Several studies have documented elevated IL-6 concentrations in follicular fluid, peritoneal fluid, or serum in women with endometriosis compared to controls (e.g., IL-6 in FF in ENDO vs. non-ENDO: 152.3 vs. ~19.9 pg/mL) [11]. IL-6 is a pleiotropic cytokine with roles in inflammation and also ovarian steroid regulation, which makes it a candidate mediator of altered folliculogenesis in ENDO. In fact, IL-6 is synthesized by granulosa/cumulus cells in the preovulatory follicle, and exerts context-dependent effects—including pro-inflammatory, regenerative, and endocrine actions—via classical (membrane IL-6R) and trans-signaling (soluble IL-6R) pathways [12]. Cortisol (active glucocorticoid) rises in intrafollicular concentrations close to ovulation and provides an endogenous anti-inflammatory signal that contributes to resolution of the ovulatory inflammatory cascade [13].

In POR or low-response settings, granulosa cells (GCs) often display reduced responsiveness to exogenous gonadotropins. In vitro studies using luteinized GCs from hypo-responders have shown deficits in steroidogenic enzyme expression and blunted response to FSH/LH stimulation [10]. These intrinsic defects may underline the failure of many standard therapeutic intensifications.

Thus, comparing ENDO and POR patients with controls (women from couples with infertility due to male factor, MF) is a compelling strategy to dissect which follicular microenvironment features—inflammatory, hormonal, paracrine—drive GC dysfunction beyond what can be explained by ovarian reserve alone. In this study, we cultured granulosa cells from ENDO, POR, and MF patients with FF collected both from their own diagnostic group and from the other two groups and assessed proliferation, morphology, and secretory activity (IL-6, cortisol, estradiol, progesterone). This comparative approach aims to elucidate how the follicular fluid environment—reflecting inflammatory, hormonal, and metabolic conditions immediately before ovulation—affects granulosa cell behavior. In doing so, we seek to better understand the mechanisms through which the FF milieu contributes to, and is itself a reflection of, the altered follicular function and infertility observed in patients with ENDO and POR.

## 2. Materials and Methods

The study was designed as a prospective, single-center investigation at the Assisted Reproductive Center, University Hospital of Padua (Italy). A total of ninety-five infertile women scheduled for IVF treatment were prospectively enrolled after providing written informed consent. The study protocol received approval from the Institutional Review Board of the University Hospital of Padua (CET-ACEV 6150/AO/24) and was conducted in accordance with the ethical principles of the Declaration of Helsinki.

Exclusion criteria included polycystic ovary syndrome, diabetes, thyroid dysfunction, amenorrhea, active or chronic infections, autoimmune disorders, severe systemic diseases, and any history of malignant or premalignant gynecological pathology. Participants were divided into three groups according to infertility etiology: male factor (MF; *n* = 30), including women whose partners had documented sperm abnormalities; endometriosis (ENDO; *n* = 38), confirmed surgically and/or by ultrasound-imaging; and poor ovarian response (POR; *n* = 27), defined as women <35 years with AMH <0.8 ng/mL and at least one prior failed IVF. POR patients were further classified using the POSEIDON criteria, with young women with low ovarian reserve (AMH < 1.2 ng/mL and/or AFC < 5) assigned to Group 3.

### 2.1. Human Follicular Fluid (FF) Processing

FF samples were obtained from IVF patients after controlled ovarian stimulation (COS) with a short GnRH antagonist protocol using follitropin-alpha (Ovaleap^®^, Theramex, London, UK) and highly purified menotropin (Meriofert^®^, IBSA Institut Biochimique SA, Lugano, Switzerland), starting on day 2 or 3 of the menstrual cycle. The total gonadotropin dose and COS duration did not differ among patients. The FSH dose was individualized based on ovarian reserve parameters (antral follicle count and AMH levels). Daily subcutaneous ganirelix (0.25 mg; Orgalutran^®^, Organon, Amsterdam, The Netherlands) was administered when at least one follicle reached 14 mm in diameter and continued until human chorionic gonadotropin (hCG) administration. Ovulation triggering was performed with 250 µg recombinant hCG (Ovitrelle^®^, Merck Serono S.p.A., Darmstadt Germany) when the leading follicle cohort exceeded 16 mm, followed 36 h later by oocyte retrieval. Follicles < 14 mm were not aspirated, in accordance with ESHRE guidelines [14].

During transvaginal oocyte retrieval, FF was collected from all punctured follicles. To minimize contamination with vaginal epithelial cells, the first aspiration tube from each patient was discarded. For each patient, FF samples from all follicles were pooled. The cellular content typically consisted of GCs, both isolated and clustered, erythrocytes, and large epithelial cells.

Collected FF was centrifuged at 200× *g* for 10 min to separate GCs [15]. The supernatant was then centrifuged at 4500× *g* for 10 min and stored at −20 °C. Upon thawing, FF underwent an additional centrifugation step (4500× *g*, 10 min), followed by sterile filtration (0.22 µm pore size) and storage at −20 °C until analysis [16].

To reduce variability in FF characteristics, samples from 2–3 women with the same infertility diagnosis (MF, ENDO, POR, respectively) were pooled [16]. To minimize potential intra-group bias, samples were selected from patients with different ages and varying stages of endometriosis. Importantly, FF pooling was performed independently of GC pooling, ensuring that group-specific differences in GCs could not be attributed to individual patient FF composition [16].

### 2.2. GCs Processing

GCs and FF samples were categorized into three groups according to the women’s infertility diagnosis. Collected GCs were washed twice by centrifugation at 200× *g* for 10 min at room temperature using culture medium (CM) containing Dulbecco’s Modified Eagle’s Medium/Nutrient Mixture F-12 (DMEM/F-12; Thermo Fisher Scientific, Waltham, MA, USA) supplemented with 8% fetal bovine serum (FBS; Thermo Fisher Scientific), 10,000 U/mL penicillin, and 10,000 µg/mL streptomycin (Thermo Fisher Scientific).

Cells were then cultured at 37 °C under standard aerobic conditions (5% CO_2_) for 24–36 h to allow viable GCs to adhere. The cultures were subsequently washed with CM to remove apoptotic or contaminating cells, and the medium was replaced with fresh CM.

After 7-days-culture, adherent cells were treated with TrypleExpress (Thermo Fisher Scientific) for 7 min at 37 °C according to the manufacturer’s indications and counted using a Makler counting chamber (SEFI Medical Instruments LTD). 5 × 10^4^ and 5 × 10^6^ cells were seeded in 12-well plates with 1 mL CM or in T25 Flask with 5 mL CM, respectively, in the presence or absence of 20% FF from each different group. To minimize variability associated with individual patient characteristics, GCs from two to three women within the same diagnostic group were pooled. Additional potential bias within each group was further reduced by selecting samples from patients with the widest possible age range and differing ENDO stages, while ensuring that GCs were never exposed to their corresponding follicular fluid (FF) during treatment [16].

Cultures were maintained at 37 °C in CM supplemented with FF obtained from women with different infertility diagnoses, generating the FF-MF, FF-ENDO, and FF-POR culture media which were subsequently replaced twice per week.

During the final five days of culture, the medium was no longer refreshed to allow accumulation of secreted factors. At the end of the incubation period, the medium was collected, designated as conditioned culture medium (CCM), and centrifuged to remove cellular debris. The resulting supernatant was then stored at −80 °C for subsequent hormone and cytokine analyses. The corresponding cells were detached as previously described and then cryopreserved in DMSO for subsequent analysis.

### 2.3. Population Replication Count

Cell proliferation was assessed by calculating the population doubling (PD) rate, as previously described [16]. Briefly, GCs were seeded at a density of 5 × 10^4^ cells per well in 12-well plates, in triplicate for each condition. On days 7, 14, and 21 of culture, cells were harvested and counted using a hemocytometer.

Population duplications at each passage were calculated as the logarithm (base 2) of the number of cells recovered at harvest (NT) minus the logarithm (base 2) of the number of cells originally plated (N0), i.e., PD = (log NT − log N0)/log 2.

### 2.4. GCs Morphology Evaluation

Phase-contrast images of GCs were acquired using a Nikon Eclipse TE2000S microscope (Nikon Corporation, Tokyo, Japan) equipped with a Cooper Surgical DC1 camera. Morphological evaluation was performed directly in 12-well tissue culture plates with a reticulated (grid) bottom (Chemglass Life Sciences, Vineland, NJ, USA), where the cells were cultured. For each experimental condition, images of at least 200 cells across 10 randomly selected fields were captured every 3 days throughout the incubation period. Cell morphology and phenotypic changes were quantitatively assessed using CellProfiler (https://cellprofiler.org/ version 4.2.8), an open-source software platform for high-throughput image analysis. Image analysis was independently conducted by two expert biologists to ensure consistency [16]. All data were subsequently collected and subjected to statistical analysis.

### 2.5. Cortisol (CORT) Quantification in FF and Conditioned Culture Medium (CCM)

Cortisol levels in FF and CCM were measured using the Beckman Coulter Access Cortisol Assay (Beckman Coulter, Brea, CA, USA), a chemiluminescent immunoassay performed on the Access 2 Immunoassay System. The assay employs competitive binding between sample cortisol and a cortisol-alkaline phosphatase conjugate, with signal intensity inversely proportional to cortisol concentration. Samples were processed according to the manufacturer’s instructions, and quality control materials were included to ensure assay reliability.

In the CCM, values are expressed as mean ± SD of ([CORT]f/[CORT]i × 1/PDs), where [CORT]f is the cortisol concentration measured at the end of the incubation period, [CORT]i is the cortisol content in the incubation medium, and PDs represents the population doubling number of the corresponding granulosa cells under those conditions.

### 2.6. Interleukin-6 (IL-6) Quantification in FF and CCM

Interleukin-6 (IL-6) levels in FF and CCM were quantified using the MAGLUMI IL-6 (CLIA) assay (Snibe Diagnostic, Shenzhen, China), a fully automated chemiluminescence immunoassay performed on the MAGLUMI series analyzers. This sandwich immunoassay employs magnetic microbeads coated with anti-IL-6 monoclonal antibodies and an ABEI-labeled anti-IL-6 monoclonal antibody. The reaction produces a chemiluminescent signal proportional to the IL-6 concentration, measured as relative light units (RLUs). Samples were processed according to the manufacturer’s instructions, and assay performance was monitored using appropriate quality control materials.

In CCM, values are expressed as mean ± SD of ([IL-6]f/[IL-6]i × 1/PDs), where [IL-6]f is the IL-6 concentration measured at the end of the incubation period, [IL-6]i is the IL-6 content in the incubation medium, and PDs represents the population doubling number of the corresponding granulosa cells under those conditions.

### 2.7. Estradiol (E2) and Progesterone (P4) Quantification in FF and CCM

E2 and P4 levels in FF and CCM were quantified using the Elecsys Estradiol III and Elecsys Progesterone III assays (Roche Diagnostics, Basel, Switzerland), respectively. Both assays are electrochemiluminescence immunoassays (ECLIA) performed on the cobas^®^ e series immunoassay analyzers. These assays utilize a sandwich principle with monoclonal antibodies and a chemiluminescent signal proportional to the analyte concentration. Samples were processed according to the manufacturer’s instructions, and assay performance was monitored using appropriate quality control materials.

In CCM, values are expressed as mean ± SD of ([Hormone]f/[Hormone]i × 1/PDs), where [Hormone]f is the hormone concentration measured at the end of the incubation period, [Hormone]i is the hormone content in the incubation medium, and PDs represents the population doubling number of the corresponding granulosa cells under those conditions

### 2.8. Follicle-Stimulating Hormone (FSH) and Luteinizing Hormone (LH) Quantification in FF

Follicle-stimulating hormone (FSH) and luteinizing hormone (LH) levels in FF were quantified using the Elecsys FSH and Elecsys LH assays (Roche Diagnostics, Basel, Switzerland), respectively. Both assays are electrochemiluminescence immunoassays (ECLIA) performed on the cobas^®^ e series immunoassay analyzers. These assays employ a sandwich principle using monoclonal antibodies, with one antibody biotinylated and the other labeled with a ruthenium complex. The chemiluminescent signal generated is proportional to the analyte concentration. Samples were processed according to the manufacturer’s instructions, and assay performance was monitored using appropriate quality control materials.

### 2.9. Total RNA Isolation and Quantitative Real Time PCR

Total RNA was isolated with the SV Total RNA Isolation System (Promega, Milan, Italy). Genomic DNA contamination was removed by on-column DNase I treatment. For RT-qPCR, 5 µg of RNA were processed using the iTaq Universal One-Step RT-qPCR Kit (Bio-Rad, Segrate, Italy). This kit contains iScript RNase H, MMLV reverse transcriptase, hot-start iTaq polymerase, and SYBR^®^ Green. Primers were designed in Primer3 and synthesized by Merck (Milan, Italy); their sequences are reported in Table S1. Reactions were run on a QuantStudio Real-Time PCR System (Thermo Fisher Scientific, Milan, Italy) for 40 cycles with a 60 °C annealing step. Expression values were normalized to β-ACTIN and processed using the ΔCT approach.

### 2.10. Statistical Analysis

Statistical analysis was performed using JMP^®^ Pro 18.0.2 (JMP Statistical Discovery LLC, SAS Institute Inc., Cary, NC, USA). Continuous data are expressed as means ± standard deviation (SD). The normality of continuous variables was assessed using the Shapiro–Wilk test. For normally distributed variables, group comparisons were performed using one-way ANOVA followed by Dunnett’s post hoc test, considering MF group as control reference. For non-normally distributed variables, the Kruskal–Wallis test was applied, followed by Dunn’s post hoc test. A *p* value < 0.05 was considered statistically significant.

## 3. Results

### 3.1. Patients’ General Parameters

The patients’ mean age was 32.1 ± 2.8 y in group MF, 35.3 ± 3.5 y in group ENDO and 33.1± 3.4 y in group POR. Only the ENDO group demonstrated a small, albeit statistically significant (*p* < 0.001) difference compared to the MF group; however, the clinical relevance was considered negligible.

Body mass index (BMI) values (23.4 ± 3.2, 23.4 ± 3.5 and 22.0 ± 2.8 in group MF, ENDO and POR, respectively) were comparable among the three groups, Follicles output rate (FORT), defined as pre-ovulatory follicle count on the day of ovulation trigger, and Follicle-to-Oocyte Index (FOI) defined as the rate between the number of oocytes retrieved at oocyte pick-up and the number of antral follicles before stimulation, showed no significant differences. Patients’ characteristics are summarized in Table 1.

### 3.2. Controlled Ovarian Stimulation (COS) Outcomes

The efficacy of the COS treatment in the three different groups was evaluated as number of mature follicles and number of oocytes and mature oocytes. The quality of the oocytes, instead, was determined by the fertilization rate, as the rate of the fecundated oocytes/of inseminated oocytes.

**Figure 1:**
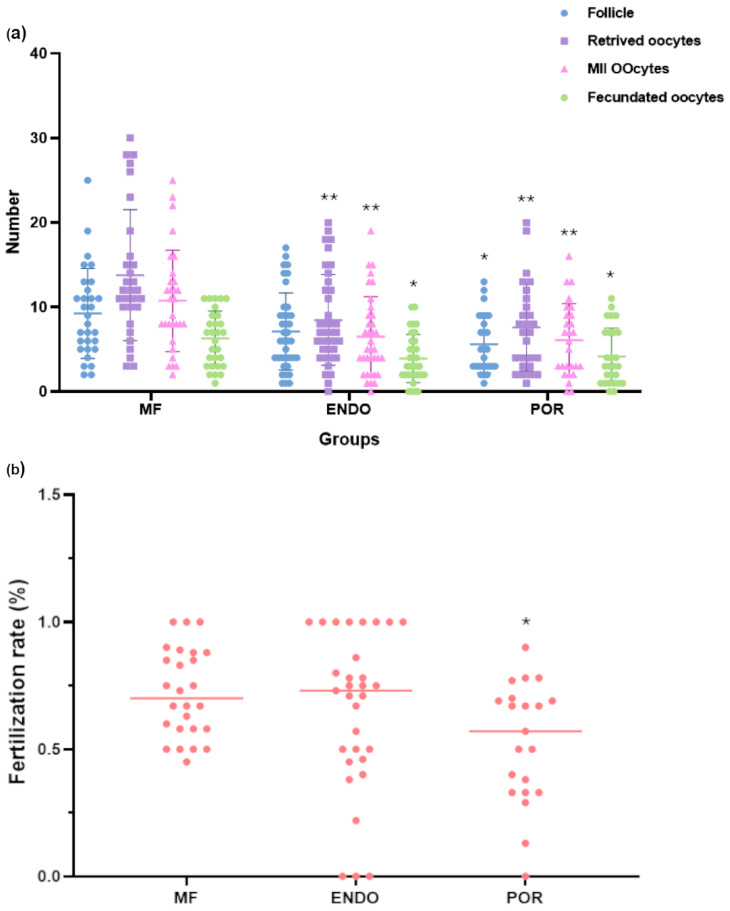
Controlled ovarian stimulation (COS) outcomes in MF (*n* = 30), ENDO (*n* = 38), and POR (*n* = 27) groups. Patients’ outcomes after COS treatment: (**a**) number of follicles, retrieved oocytes, MII oocytes (mature oocytes) and fecundated oocyte and (**b**) the fertilization rate for each group were evaluated by one-way ANOVA followed by Dunnett’s post hoc test (comparison vs. MF group), or with Kruskal-Wallis test followed by Dunn’s test in case of non-normal data. Pink circles represent individual fertilization rate values, with the median shown as a line * *p <* 0.05; ** *p <* 0.01.

Patients’ mean outcomes of the COS treatment are summarized in Table 2, as mean ± SD and in Figure 1 as individual values characterizing each patient.

COS outcomes varied significantly across the three groups (Figure 1). Compared with MF patients, both ENDO and POR groups showed a significant reduction in the number of retrieved oocytes and mature (MII) oocytes (*p* < 0.01 for ENDO; *p* < 0.01 for POR). The number of follicles and fecundated oocytes was also lower, with ENDO patients displaying moderate reductions (*p* < 0.05 for fecundated oocytes) and POR patients showing the most pronounced impairment (*p <* 0.05–0.01).

Fertilization rate analysis highlighted a distinct pattern, with ENDO patients showing values comparable to MF, while POR patients exhibited a modest but significant reduction (*p* < 0.05). Overall, the findings suggest that ENDO is mainly associated with a reduced oocyte yield, whereas POR is characterized by a lower number of retrieved oocytes and a tendency toward reduced fertilization efficiency.

### 3.3. FF-Related Hormonal Level and Morphological Parameters of GCs in Patients’ Follicular Fluid (FF)

**Figure 2:**
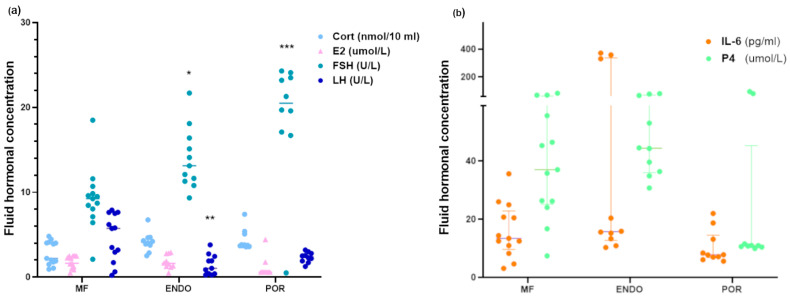
Hormonal and IL-6 parameters were analyzed in follicular fluid samples collected at oocyte pick-up. The content of hormones and the cytokine IL-6 was assessed as described in the Methods; (**a**) Hormone concentrations; (**b**) IL-6 and P4 levels. Because most datasets deviated from normality according to the Shapiro–Wilk test, statistical analysis was conducted using the Kruskal–Wallis test followed by Dunn’s multiple-comparisons test (vs. MF group). * *p* < 0.05; ** *p* < 0.01; *** *p* < 0.001. Data represent individual follicular fluid samples (*n* = 9–14 per group, depending on parameter). Cort: cortisol; E2: estradiol; FSH: follicle-stimulating hormone; LH: luteinizing hormone; P4: progesterone.

Follicular fluid analysis revealed significant differences in hormonal and cytokine concentrations among the study groups (MF, ENDO, POR) (Figure 2).

For steroid hormones, FSH levels were markedly higher in the POR group and, to a lesser extent, in the ENDO group compared to MF (*p* < 0.001 and *p* < 0.05, respectively). LH concentrations were significantly reduced in the ENDO group (*p* < 0.01), while no significant difference was detected for the POR group. No significant variations in E2 concentrations or P4 levels were found among groups. CORT concentrations showed no significant differences between groups in the nonparametric analysis. Regarding inflammatory and luteal markers, IL-6 levels were not significantly different among groups according to Dunn’s post hoc test, despite a trend toward higher values in the ENDO group.

These results slightly differ from an initial parametric analysis (one-way ANOVA followed by Dunnett’s test), in which CORT and IL-6 appeared significantly increased in the ENDO group. Given the non-normal distribution of these variables, nonparametric testing (Kruskal–Wallis plus Dunn’s test) was considered more appropriate, and its outcomes were taken as primary. The direction of the observed effects remained consistent, although statistical significance was attenuated.

The phenotypes of the three patient groups were evaluated based on the main morphological characteristics of granulosa cells (GCs) collected from follicular fluid at pickup. GCs were classified into three subpopulations: follicle epithelial-like GCs (FELGCs), fibroblast-like GCs (FLGCs), and undifferentiated GCs (uGCs) [16].

FELGCs appeared as large, flat, rough cells lacking filaments or protrusions. They adhered readily to culture plates or flasks but failed to re-adhere after passage. FLGCs exhibited an elongated, tapered shape resembling fibroblasts, with long extensions establishing contact with neighboring cells. They survived and re-adhered efficiently after passage. uGCs were small, irregular, bean-shaped cells surrounded by multiple protrusions; like FLGCs, they also survived and re-adhered after passage.

**Figure 3:**
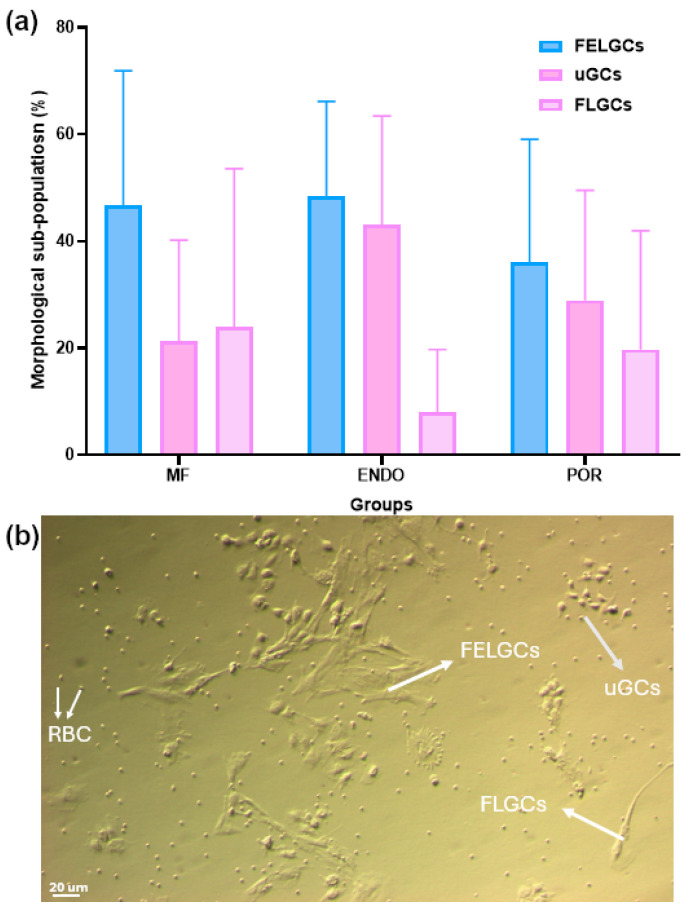
Comparison of morphology-related sub-populations. Cells collected at oocyte pick-up were cultured for 24–36 h, washed in CM to remove apoptotic cells, and subsequently incubated in CM for an additional 4–5 days to allow stabilization and development. (**a**) Morphological evaluation was then performed as described in the Methods. Data represent the mean ± SD of 19–27 samples per group. (**b**) GCs sub-population morphology. Contrast-phase images showing morphologically distinct cells present at the early stage of incubation after oocyte pick-up. FELGC: Follicle Epithelial-Like Granulosa Cells; uGCs: undifferentiated Granulosa Cells; FLGCs: Fibroblast-Like Granulosa Cells.

Morphological evaluation of GCs (Figure 3) revealed marked variability in the distribution of these subpopulations across all groups. FELGCs and uGCs were similarly represented, whereas the proportion of FLGCs showed a tendency toward lower values in the ENDO group compared with MF (8.0 ± 11.7% vs. 24.0 ± 29.6%), although this difference did not reach statistical significance according to the Kruskal–Wallis test followed by Dunn’s post hoc analysis.

### 3.4. Experimental Outcomes of GCs Cultured in Culture Medium Supplemented with the Three Different FF

Prior to evaluating the impact of the three FF treatments on granulosa cells, we characterized the composition of each culture medium (CM supplemented with FF) by measuring its hormonal and IL-6 content.

Analysis of hormone and cytokine content in the culture media (Table 3) revealed overall variability among groups. CORT concentrations tended to be higher in the POR and ENDO groups compared with MF, although these differences did not reach statistical significance in the Kruskal–Wallis analysis. Similarly, IL-6, E2, and P4 levels showed group-related trends, with higher mean values in ENDO than in MF, but without significant pairwise differences. Notably, FSH levels remained significantly elevated in the POR group compared with MF (*p* < 0.001), consistent with the more intensive COS regimens applied in these patients. LH concentrations were comparable among groups, although a tendency to be lower was observed in ENDO group.

#### 3.4.1. Population Doubling (PDs)

PDs were evaluated for each group in the three different treatments (Table 4 and Figure S1).

**Figure 4:**
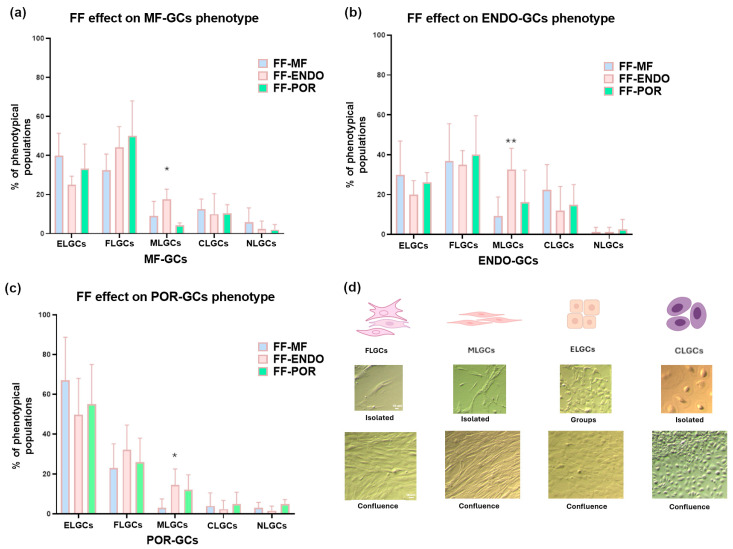
Effects of follicular fluid treatments on GC morphological sub-populations. GCs, collected at oocyte pick-up from MF (**a**), ENDO (**b**) or POR (**c**) groups, were first incubated in control medium (CM) for 4–5 days to allow stabilization and development, then divided into three samples and cultured for 3 weeks in media supplemented with FF-MF, FF-ENDO, or FF-POR. Morphological assessment was performed by analyzing 10 fields per plate as described in Methods section. A total of 500 cells per sample were evaluated and classified into ELGCs: epithelial-like granulosa cells; FLGCs: fibroblast-like granulosa cells; MLGCs: muscle-like granulosa cells; CLGCs: chondroblast-like granulosa cells; NLGCs: neuronal-like granulosa cells. (**d**) Contrast-phase images showing morphologically distinct GC subpopulations at early culture stages (isolated cells or small clusters) and at confluence. Scale bar = 10 μm. Data are expressed as mean ± SD (%) from six independent experiments per group. Statistical analysis was performed using one-way ANOVA followed by Dunnett’s post hoc test (comparison vs. MF group) or with Kruskal-Wallis test followed by Dunn’s test in case of non-normal data. * *p* < 0.05; ** *p* < 0.01 vs. MF group.

When comparing granulosa cells (GCs) across patient groups (MF, ENDO, POR), no significant differences in proliferation were observed when the same follicular fluid (FF) was applied (Figure 4a). For instance, in the presence of FF-MF, GCs from all three groups (MF, ENDO, POR) displayed very similar replication rates, with nearly identical population doubling (PD) values. The same pattern was evident for FF-ENDO and FF-POR, where GCs replicated at comparable rates regardless of their patient group of origin. Importantly, overall proliferation was consistently higher in the presence of FF-ENDO and moderately increased with FF-POR, compared to FF-MF. This indicates that the replication rate depends on the source of the FF, rather than on the origin of the GCs.

This interpretation is further supported by the within-group analysis (Figure 4b, Table 4). MF-derived GCs showed significantly greater proliferation when cultured with FF-ENDO (2.22 ± 0.43 vs. 1.28 ± 0.15 with FF-MF, *p* < 0.001) and, to a lesser extent, with FF-POR (1.61 ± 0.23, *p* < 0.05). ENDO-derived GCs proliferated robustly in FF-ENDO (2.22 ± 0.42), but their growth was significantly reduced in FF-MF (1.30 ± 0.11, *p* < 0.001), suggesting a normalization effect. POR-derived GCs also replicated more efficiently in FF-ENDO (2.47 ± 0.41 vs. 1.28 ± 0.15 with FF-MF, *p* < 0.001) and showed a moderate increase with FF-POR (1.80 ± 0.30 vs. 1.40 ± 0.24).

Taken together, these findings demonstrate that granulosa cell proliferation is primarily driven by the type of follicular fluid, with FF-ENDO exerting the strongest stimulatory effect across all GC groups, followed by FF-POR, and lowest with FF-MF.

#### 3.4.2. Morphological Alterations

The FF treatment affected not only the replication rate of GCs in culture but also their morphological differentiation. The large, flat epithelial-like granulosa cells (ELGCs), which closely resembled the follicular epithelial layer, completely disappeared during culture and were lost after passaging procedures (cell detachment and reseeding). In contrast, the undifferentiated GC population (uGCs), initially characterized by a round and poorly defined morphology, displayed a remarkable ability to acquire distinct phenotypes over time. These included long, slender muscle-like GCs (MLGCs), round and compact chondrocyte-like GCs (CLGCs), and cuboid epithelial-like GCs (ELGCs), indicating that FF exposure supports the plasticity and morphological diversification of GCs in vitro.

Analysis of GC morphology (Figure 4) showed treatment-specific and source-dependent effects when each condition was compared to the FF-MF baseline in MF-GCs. In MF-GCs, in fact, exposure to FF-ENDO increased the proportion of muscle-like GCs (MLGCs) relative to FF-MF, indicating that endometriosis-derived follicular fluid can promote a muscle-like phenotype even in GCs from morphologically fertile donors. On the contrary, in ENDO-GCs, FF-MF reduced the MLGC fraction compared with FF-ENDO, consistent with a partial restorative effect of MF-derived fluid on the disease-associated MLGC enrichment. In POR-GCs, FF-MF almost completely suppressed the MLGC subpopulation compared with FF-ENDO and, to a lesser extent, compared with FF-POR, showing that MF-derived fluid strongly counteracts MLGC prevalence in poor-responder cells. By contrast, both FF-ENDO and FF-POR tended to maintain higher MLGC frequencies in POR-GCs. Across all three GC sources, the proportions of epithelial-like (ELGCs), fibroblast-like (FLGCs), chondroblast-like (CLGCs), and neuronal-like (NLGCs) cells showed no consistent, treatment-dependent shifts. Taken together, these data suggest that MLGCs are the most treatment-sensitive subpopulation: FF-ENDO promotes MLGC enrichment in MF and ENDO origins, whereas FF-MF exerts a normalizing/suppressive effect on MLGCs in disease-derived GCs—a particularly strong effect in POR-GCs.

#### 3.4.3. Analysis of CORT, IL-6, E2 and P4 in the Conditioned Culture Medium (CCM)

**Figure 5:**
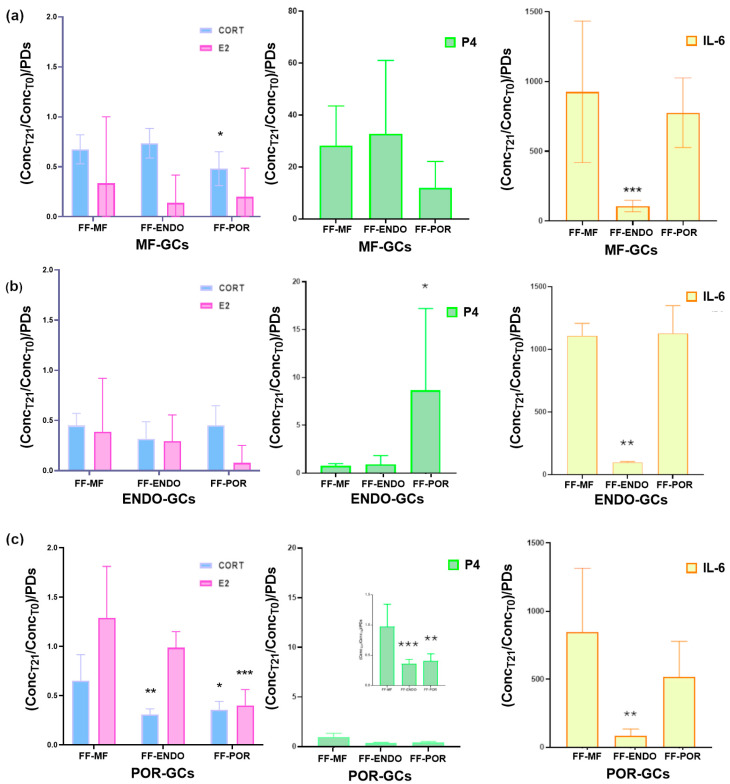
Modulation of hormone and cytokine levels in culture supernatants of granulosa cells (GCs) treated with FF. GCs from MF (**a**), ENDO (**b**), and POR (**c**) patients were cultured for 3 weeks in the presence of FF-MF, FF-ENDO, or FF-POR, as described in Methods. Five days before the end of culture, medium replacement was stopped, and conditioned culture medium (CCM) was collected, centrifuged, and analyzed for cortisol (CORT, blue), estradiol (E2, pink), progesterone (P4, green), and IL-6 (yellow) content. Data were normalized to the corresponding baseline concentrations in each supplemented medium (FF-MF, FF-ENDO, FF-POR) and to the relative PDs in the same conditions, and expressed as relative consumption or release ([Hormone or IL-6]f/[Hormone or IL-6]i × 1/PDs). Values represent mean ± SD from at least six independent experiments per group. Statistical analysis was performed by one-way ANOVA followed by Dunnett’s post hoc test (comparison vs. MF group) or with Kruskal-Wallis test followed by Dunn’s test in case of non-normal data. * *p* < 0.05; ** *p* < 0.01; *** *p* < 0.001.

To further investigate the impact of FF on GC function, we analyzed the culture supernatants collected at the end of the treatments. These samples reflect the hormonal and cytokine output of the cells and provide valuable information on how different FF sources modulate GC secretory activity (Figure 5).

Analysis of the conditioned media revealed that FF treatment differentially modulated hormone consumption and cytokine release in GCs depending on their origin.

Hormones and IL-6 secretion were quantified as the ratio between the final concentration detected in the conditioned culture medium (CCM) and the initial concentration present in the follicular fluid–supplemented medium (FF-CM) (Table 4). Thus, values represent the relative hormonal and IL-6 production by granulosa cells compared with the baseline provided by the added FF.

Using this approach, MF-GCs (Figure 5a) showed consumption of both CORT and, to an even greater extent, E2 under all culture conditions, indicating that MF-GCs actively utilize CORT during in vitro development. Interestingly, P4 was actively produced under all conditions, with comparable levels in FF-MF and FF-ENDO, but nearly 50% lower in the presence of FF-POR. Also, MF-GCs showed a dramatic induction of IL-6, with increases approaching 1000-fold when cultured in FF-MF or FF-POR, whereas in FF-ENDO the induction was much lower, about 100-fold over baseline and roughly ten-fold less than in the other two conditions.

ENDO-GCs (Figure 5b) followed a similar trend, producing large amounts of IL-6 in FF-MF and FF-POR, but showing markedly reduced secretion in FF-ENDO. In addition, P4 accumulation was significantly enhanced only in the presence of FF-POR, although to nearly half the amount observed in MF-GCs under the same condition. CORT and E2 consumption remained unchanged across treatments and were comparable to the levels measured in MF-GCs.

POR-GCs (Figure 5c) also secreted abundant IL-6 under FF-MF and FF-POR conditions, with increases of several hundred-fold compared with baseline, while their output was strongly suppressed in FF-ENDO. Overall, these results demonstrate that although all GC types are capable of producing IL-6 far above baseline, the presence of FF-ENDO consistently restrains this response across groups, in sharp contrast to FF-MF and FF-POR, which strongly promote IL-6 release.

Overall, these findings indicate that, compared to FF-MF, FF-ENDO and FF-POR exert distinct modulatory effects on GC secretory activity. ENDO-derived fluid consistently suppressed IL-6 release, pointing to an anti-inflammatory effect, while POR-derived fluid strongly reduced estradiol and progesterone levels, highlighting a potential impairment of steroidogenic activity in GCs exposed to POR microenvironmental cues.

**Figure 6:**
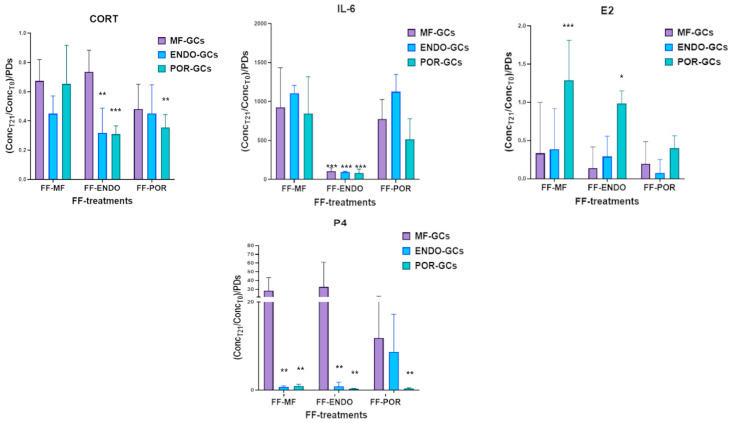
Modulation of hormone and cytokine levels in culture supernatants of granulosa cells (GCs) treated with FF. GCs from MF (violet), ENDO (light blue), and POR (green) patients were cultured for 3 weeks in the presence of FF-MF, FF-ENDO, or FF-POR, as described in Methods. Five days before the end of culture, medium replacement was stopped, and conditioned culture medium (CCM) was collected, centrifuged, and analyzed for cortisol (CORT), estradiol (E2), progesterone (P4), and IL-6 content. Data were normalized to the corresponding baseline concentrations in each supplemented medium (FF-MF, FF-ENDO, FF-POR) and to the relative PDs in the same conditions, and expressed as relative consumption or release ([Hormone or IL-6]f/[Hormone or IL-6]i × 1/PDs). Values represent mean ± SD from at least six independent experiments per group. Statistical analysis was performed by one-way ANOVA followed by Dunnett’s post hoc test or with Kruskal-Wallis test followed by Dunn’s test in case of non-normal data (comparison vs. MF-GCs in FF-MF). * p < 0.05; ** *p* < 0.01; *** *p* < 0.001.

When directly comparing the three GC groups for each analyte (Figure 6), both CORT and E2 appeared to be predominantly consumed rather than produced in vitro, although to different extents. MF-GCs consumed less CORT in all follicular fluid conditions compared to ENDO-GCs and POR-GCs. With respect to E2, almost complete consumption was observed in both MF-GCs and ENDO-GCs, whereas POR-GCs showed a slight but consistent net expression in all conditions.

A different scenario emerged for IL-6 and progesterone (P4). IL-6 was actively produced at very high levels by MF-GCs and POR-GCs, reaching 926.50 ± 506.71 and 776.56 ± 249.14 in FF-MF and FF-POR, respectively, for MF-GCs, and 845.69 ± 471.28 and 516.56 ± 262.45 times the baseline levels in FF-MF and FF-POR, respectively, for POR-GCs. In sharp contrast, in the presence of FF-ENDO, none of the three GC groups expressed IL-6 levels higher than 100, suggesting that endometriosis-associated follicular fluid strongly restricts IL-6 production.

Progesterone expression was largely restricted to MF-GCs, which reached 28.28 ± 15.21 and 32.70 ± 28.32 times the baseline level in FF-MF and FF-ENDO, respectively, while only 11.86 ± 10.27 was observed in FF-POR. No meaningful P4 production was detected in the other GC groups, except for ENDO-GCs incubated with FF-POR, which induced P4 expression at 8.66 ± 8.55 times the baseline level, albeit with a very high variability across samples. Overall, the standard deviations were relatively high, indicating a degree of variability across samples.

#### 3.4.4. Gene Expression Analysis in GCs Exposed to the Three Different FF-Media

**Figure 7:**
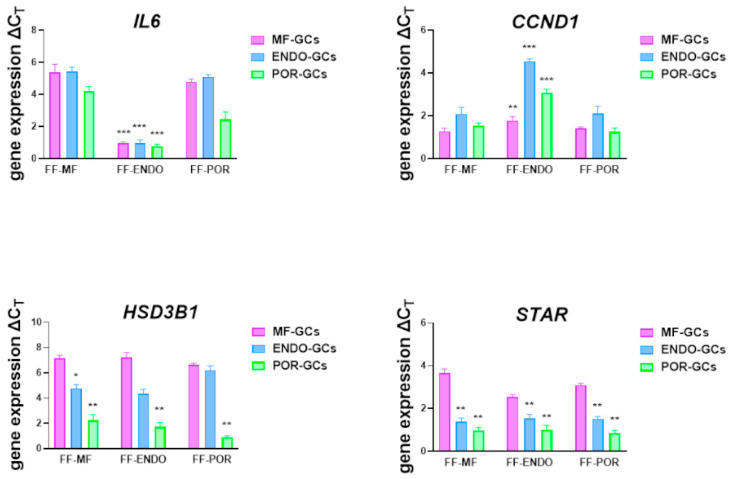
Modulation of gene expression in GCs treated with the three different FF-media. GCs from MF (violet), ENDO (light blue), and POR (green) patients were cultured for 3 weeks in the presence of FF-MF, FF-ENDO or FF-POR as described in Methods. At the end of culture, cells were collected and subjected to total RNA extraction. Quantitative real time PCR was performed to evaluate the gene expression levels of IL-6, (*IL-6*), cyclin D1 (*CCND1*), hydroxy-Delta-5-steroid dehydrogenase, 3 Beta- and Steroid Delta-Isomerase 1 (*HSD3B1*) and steroidogenic acute regulatory (*STAR*). Data were normalized to the expression of *β-ACTIN* gene as the internal reference and calculated using the ΔCT method. Values are reported as mean ± SEM from at least six independent experiments per group. Statistical analysis was performed by one-way ANOVA followed by Bonferroni’s multicomparison test. * denotes *p* < 0.05; ** denotes *p* < 0.01; *** denotes *p* < 0.001.

Analysis of gene expression in the GCs that produced the previously described CCM—characterized for its hormone and IL-6 contents showed distinct inflammatory, proliferative, and steroidogenic expression patterns (Figure 7).

IL-6 expression was markedly upregulated in both MF-GCs and ENDO-GCs when cultured with FF-MF or FF-POR. In contrast, exposure to FF-ENDO significantly reduced IL-6 expression, leading to a partial inhibition also observed in POR-GCs. Conversely, POR-GCs exhibited moderately decreased IL-6 levels in FF-MF, an effect that became more pronounced in the presence of FF-POR.

Consistent with their previously described PD profile, all three cell types showed increased CCND1 expression, indicating enhanced proliferation, when exposed to FF-ENDO. Steroidogenic function, measured by HSD3B1 and STAR levels, rose significantly only in MF-GCs in all the conditions. In contrast, POR-GCs exhibited reduced steroidogenic gene expression under every condition tested, and ENDO-GCs showed a similar reduction, especially when treated with FF-ENDO.

## 4. Discussion

**Figure 8:**
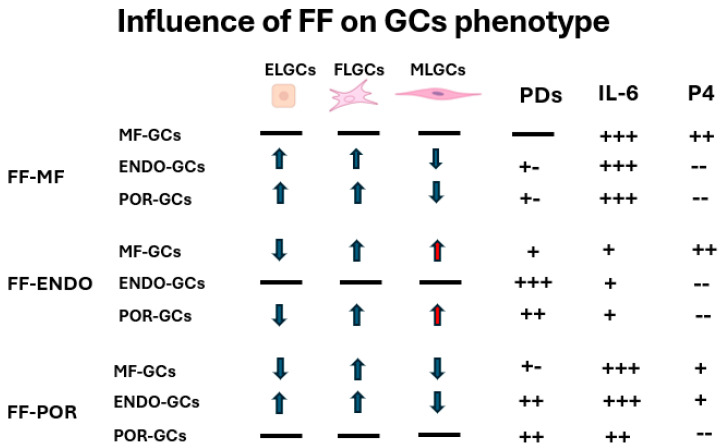
Schematic summary of the principal effects exerted by each type of FF on GCs derived from the three study groups. Arrows indicate the direction of morphological changes (increase or decrease, with red marking potential detrimental effect) whereas the solid line “**___**” represents the baseline value. The PDs are expressed as relative to the baseline (solid line): “**+++**” denotes the highest observed increase, whereas “**+ -**” indicates the lowest, and +, or ++ intermediate values. The concentrations of IL-6 and progesterone (P4) measured in the CCM are depicted using plus signs: “**+++**” corresponds to the highest levels observed, while “**--**” indicates no detectable expression.

The present study demonstrates that although granulosa cells (GCs) initially exhibited similar morphological distributions into the three subpopulations (FELGCs, uGCs, and FLGCs) at the time of oocyte pick-up, their subsequent behavior was largely dictated by the follicular fluid (FF) microenvironment. FF composition emerged as the primary driver of GC proliferation, morphology, and—most importantly—secretory activity, while the cellular origin modulated the extent of these responses. FF-ENDO exerted the strongest proliferative stimulus and promoted enrichment of muscle-like GCs, whereas FF-POR significantly impaired steroidogenic activity. Secretory outcomes (CORT, E2, P4 and IL-6) reflected a complex interplay between FF composition and GC origin: MF- and POR-derived GCs secreted very high levels of IL-6 in FF-MF and FF-POR conditions, whereas FF-ENDO consistently suppressed IL-6 release across all GC types. CORT and E2 were generally consumed in vitro, with POR-GCs showing the lowest E2 consumption, while progesterone production was largely restricted to MF-GCs, except for a variable induction of P4 in ENDO-GCs cultured with FF-POR. These findings, summarized in the Figure 8, indicate that pathological FF signatures (particularly those associated with endometriosis and poor ovarian response) reprogram GC secretory behavior in distinct ways with potential consequences for follicle health and oocyte competence [18,19].

The induction of P4 production in ENDO-GCs by FF-POR represents a particularly intriguing observation. This phenomenon may reflect a compensatory mechanism, whereby ENDO-derived cells respond to the hormonally impoverished POR microenvironment by upregulating luteinization or P4-synthesis pathways. One could speculate that FF-POR contains a specific cue—or acts through a receptor-mediated mechanism—that selectively triggers P4 synthesis in ENDO-GCs but not in other GC types. Indeed, this response was absent in POR-GCs, which appear intrinsically deficient in steroidogenic function, and only weakly present in MF-GCs. Most intriguingly, the effect appears FF-specific: while FF-MF broadly supported P4 expression across all GC origins, FF-POR selectively induced it only in ENDO-GCs. This aligns with evidence that IL-6 signalling can modulate luteal progesterone output [20] and that POR-GCs exhibit intrinsic defects in steroidogenesis, including reduced Steroidogenic Acute Regulatory protein (StAR) and 3ß-hydroxysteroid dehydrogenase (3ßHSD) activity [21].

In a previous study [16], we observed that FF-ENDO induced a higher expression of connexin 43 (Cx43) compared with FF-MF and hypothesized that this increase in intercellular junctions could enhance the exchange of nutrients and signaling molecules. Our current findings are consistent with this interpretation: FF-ENDO strongly stimulated GC proliferation and reshaped secretory activity across all GC groups. Indeed, Cx43-mediated communication has been reported as essential for granulosa cell proliferation, follicular development, and oocyte competence [22]. External factors, including TGF-β1 or metabolic/inflammatory cues, can further upregulate Cx43 in human granulosa cells [23], suggesting that the altered composition of FF-ENDO may act through similar pathways.

Although cumulus cells (CCs) were not included in this work, their intimate connection with the oocyte suggests that similar, FF-dependent modulation of Cx43 and gap-junctional activity may occur in the cumulus compartment, influencing cumulus expansion and oocyte maturation. This hypothesis will be the focus of our future studies.

Interestingly, the unbalanced composition of the phenotypic sub-populations induced by FF-ENDO and, though to a lesser extent, by FF-POR, may indicate a drift toward dysregulated luteinization. Under physiological conditions, luteinization requires not only a shift in steroidogenic profile but also profound tissue remodeling, including angiogenesis and restructuring of the extracellular matrix (ECM). After the LH/hCG surge, the granulosa layer becomes vascularized as endothelial cells from the theca invade [24] and the newly formed blood vessels must stabilize to support the metabolic demand of progesterone synthesis. ECM remodeling via precise balance of matrix metalloproteinases (such as collagenases, gelatinases, stromelysins) and tissue inhibitors of metalloproteinases is essential for follicular rupture and corpus luteum formation [25] and components like fibronectin, laminin, and integrin-mediated signaling promote morphological changes in GCs and induce maximal progesterone production via Hsd3b activity [26]. In addition, culture studies show that GC progesterone output is sensitive to the ECM context (solid vs. liquid matrices, collagen/laminin/fibronectin coatings) underscoring that ECM architecture is not just scaffolding but an active modulator of luteal function [27].

Experimental studies further highlight the mechanistic link between ECM remodeling and granulosa cell luteinization. For instance, fibronectin–integrin interactions are required for cumulus expansion and maximal induction of Hsd3b, a key enzyme for progesterone biosynthesis [28]. Similarly, gene expression profiling in bovine GCs has shown that enhanced cell–cell and cell–matrix contacts promote a steroidogenic shift characterized by reduced estradiol and increased progesterone output [29]. Moreover, luteal fibroblast-like cells actively participate in collagen production under the influence of fibroblast growth factor, linking fibroblast-like morphology to tissue remodeling capacity during luteal development or regression [30].

Taken together, our findings suggest that the divergent behavior of GCs cultured in FF from different patient groups reflects the presence of distinct bioactive factors that modulate GC function at multiple molecular levels. Both ENDO- and POR-derived FF appear to influence GC proliferation, morphology, and secretory activity, albeit to different extents, suggesting a gradient of biological activity related to the composition of the FF microenvironment. Previous studies have reported that FF from altered ovarian conditions contains altered levels of inflammatory cytokines (IL-1β, TNF-α, IL-6), oxidative stress mediators, and altered lipid or iron metabolism, all of which can activate intracellular pathways such as NF-κB, JAK/STAT3, and MAPK, leading to changes in IL-6 expression and steroidogenic enzyme regulation (STAR, CYP11A1, HSD3B) [31,32,33]. Furthermore, accumulating evidence indicates that FF contains extracellular vesicles (EVs)—including exosomes and microvesicles—that transport regulatory molecules such as microRNAs, long non-coding RNAs, mRNAs, and proteins capable of modulating gene expression and cell-cycle progression in recipient cells [34,35]. Variations in the abundance or cargo of these EVs among FF sources could therefore contribute to the differential GC responses observed in our cultures, by reprogramming intracellular signaling and metabolic pathways. Overall, both soluble factors and EV-mediated molecular transfer likely cooperate to shape GC behavior, accounting for the distinct proliferative patterns, morphological features, and variable IL-6 and progesterone secretion profiles identified in our study. Future studies in larger and stratified patient populations, integrating molecular and multi-omics approaches, will be crucial to identify the causal drivers of the observed CCMdiversity and to better define therapeutic interventions aimed at restoring optimal follicular and luteal function in ART settings

In addition, these findings suggest that the fibroblast-like and muscle-like phenotypes observed in our granulosa cell cultures may predispose to abnormal ECM turnover and incomplete luteinization. Such alterations could compromise the establishment of a fully functional corpus luteum and thereby limit progesterone synthesis. This framework offers a potential explanation for our observation that progesterone production was confined largely to MF-GCs, with only a partial and inconsistent induction in ENDO-GCs under FF-POR, while POR-GCs remained essentially unable to mount a steroidogenic response.

To our knowledge, this is the first study in which GCs and FF obtained from women with different reproductive conditions (MF, POR, and ENDO) were combined in a reciprocal cross-over experimental design. Previous investigations have generally addressed isolated FF components—such as cytokines [36], extracellular vesicles/exosomes [37], or altered iron and lipid metabolism [31,38]—and described limited or unidirectional effects on GC function. In contrast, by systematically interchanging FF and GCs from distinct clinical backgrounds, we show that the FF composition influence GC proliferation, morphology, and IL-6 and progesterone secretion. These findings indicate that the interaction between humoral and cellular follicular components is pathology-dependent and support the view that alterations in the FF milieu can actively shape GC behavior and, ultimately, follicular competence.

## 5. Conclusions

Understanding what influences not only the proper development of the ovarian follicle and, consequently, the maturation of a competent oocyte, but also the correct luteinization of the residual follicle after ovulation, is fundamental for selecting the most effective therapeutic strategies to achieve successful outcomes in assisted reproductive technologies (ART). The present findings highlight the potential clinical relevance of FF composition in determining granulosa-cell and, consequently, follicular function. A better understanding of how distinct FF milieus modulate GC proliferation, cytokine release, and steroidogenesis could be useful to identify biochemical or extracellular-vesicle–based markers of follicular competence, improving oocyte selection and cycle management in ART. Future studies should aim to characterize the molecular mediators responsible for these effects and to assess whether modulation of the follicular microenvironment—through tailored stimulation, antioxidant or anti-inflammatory interventions, or optimized in-vitro culture supplements—can enhance oocyte quality and clinical outcomes.

The present study demonstrates that granulosa cell proliferation, morphology, and secretory activity are primarily dictated by the composition of follicular fluid (FF) rather than by the intrinsic origin of the GCs themselves. In particular, our data indicate that the high levels of IL-6 and FSH that characterize FF-ENDO and FF-POR may be not sufficient to sustain robust P4 synthesis. Instead, P4 production remained largely a prerogative of MF-derived FF, suggesting that additional, yet unidentified, microenvironmental cues are required to activate or maintain the steroidogenic machinery. This highlights that progesterone deficiency in POR and ENDO conditions cannot be explained by gonadotropin or cytokine levels alone but likely reflects more complex alterations in the follicular niche.

We acknowledge that in vitro culture models, even when supplemented with FF, cannot fully reproduce the dynamic paracrine and endocrine interactions of the in vivo ovary. Moreover, the relatively small cohort size and inter-patient variability (reflected by high SDs) limit the generalizability of our conclusions. Future studies in larger and stratified patient populations, integrating molecular and multi-omics approaches, will be crucial to identify the causal drivers of the observed CCM diversity and to better define therapeutic interventions aimed at restoring optimal follicular and luteal function in ART settings.

## Figures and Tables

**Table 1:** Patients’ parameters. Total FSH dose (expressed in international units) administered during COS, Age and Body Mass Index (BMI), were considered for the evaluation of ovarian sensitivity. Additional parameters included Follicle Output Rate (FORT; defined as the ratio of pre-ovulatory follicles on the day of ovulation trigger [1]), and Follicle-to-Oocyte Index (FOI; values > 0.50 indicating a normal number of oocytes retrieved at oocyte pick-up) [17].

	MF *n* = 30	ENDO *n* = 38	POR *n* = 27
Total FSH (IU)	2135.0 ± 732.7	2617.1 ± 829.5 *	2630.8 ± 663.0 *
Age (years)	32.1 ± 2.8	35.3 ± 3.5 ***	33.1± 3.4
BMI (kg/m^2^)	23.4 ± 3.7	23.4 ± 3.5	22.0 ± 2.8
FORT	1. 5 ± 1.4	1.4 ± 0.8	1.1 ± 0.7
FOI	1.7 ± 1.0	1.5 ± 1.3	1.6 ± 1.0

*** *p* < 0.001, * *p* < 0.05 vs. MF, ANOVA followed by Dunnett’s test, or Kruskal–Wallis test followed by Dunn’s test for non-normal data.

**Table 2:** IVF outcomes of the three patient groups. Data are presented as mean ± SD for the parameters evaluated in each group. Fertilization rate was calculated as the ratio of fecundated oocytes to the number of inseminated oocytes and mature oocytes (metaphase II, MII).

Parameters	Patients		
	MF *n* = 30	ENDO *n* = 38	POR *n* = 27
**Follicle number**	9.2 ± 5.3	7.1 ± 4.5	5.6 ± 3.4 *
**Retrieved oocytes**	13.8 ± 7.8	8.5 ± 5.4 **	7.6 ± 5.2 **
**MII oocytes**	10.7 ± 6.0	6.5 ± 4.7 **	6.1 ± 4.3 **
**Fecundated oocytes**	6.3 ± 3.3	3.9 ± 2.8 *	4.2 ± 3.5 *
**Fertilization rate**	0.7 ± 0.2	0.7 ± 0.3	0.5 ± 0.2 *

** *p* < 0.01, * *p* < 0.05 vs. MF, ANOVA followed by Dunnett’s test, or Kruskal–Wallis test followed by Dunn’s test for non-normal data.

**Table 3:** Hormones and IL-6 content in the culturing media. The media are referred to as follows: FF-MF, CM supplemented with FF from the MF group; FF-ENDO, CM supplemented with FF from the ENDO group; and FF-POR, CM supplemented with FF from the POR group. Data are presented as mean ± SD (*n* = 5–13 experiments). Statistical analysis was performed using one-way ANOVA followed by Dunnett’s post hoc test (comparisons with the MF group) for normally-distributed FSH values, and with Kruskal-Wallis test followed by Dunn’s test for remaining non-normal data. *** *p* < 0.001.

	CORT (nmol/L)	IL-6 (pg/mL)	E2 (μmol/L)	P4 (μmol/L)	FSH (IU/L)	LH (IU/L)
FF-MF	56.50 ± 28.19	3.19 ± 1.84	0.34 ± 0.15	8.28 ± 4.41	1.80 ± 0.72	0.72 ± 0.58
FF-ENDO	72.70 ± 15.10	36.83 ± 37.17	0.69 ± 0.38	12.59 ± 6.39	2.64 ± 0.76	0.22 ± 0.20
FF-POR	91.19 ± 26.48	2.24 ± 1.21	0.33 ± 0.35	7.83 ± 7.96	3.32 ± 0.59 ***	0.62 ± 0.73

**Table 4:** Population duplications (PDs) of granulosa cells (GCs) from different groups cultured in the three follicular fluids. Data are presented as mean ± SD (*n* = 9). Statistical analysis was performed using one-way ANOVA followed by Dunnett’s post hoc test, with comparisons made against the MF group. *** *p* < 0.001, * *p* < 0.05.

	PDs	PDs	PDs
	FF-MF	FF-ENDO	FF-POR
MF-GCs	1.28 ± 0.15	2.22 ± 0.43 ***	1.61 ± 0.23 *
ENDO-GCs	1.3 ± 0.11	2.22 ± 0.42 ***	1.54 ± 0.22
POR-GCs	1.4 ± 0.24	2.47 ± 0.41 ***	1.8 ± 0.30

## Data Availability

The original contributions presented in this study are included in the article/Supplementary Materials. Further inquiries can be directed to the corresponding author.

## Supplementary Materials

The following supporting information can be downloaded at: https://www.mdpi.com/article/10.3390/biom15121646/s1. Figure S1. Population duplications (PDs) of the cells of each group (MF-GCs, ENDO-GCs and POR-GCs) in the culture medium supplemented with follicular fluid of MF (FF-MF), ENDO (FF-ENDO) and POR (FF-POR). (a) Analysis of PDs obtained by each group of GCs in FF-MF (upper) FF-ENDO (centre) and FF-POR (lower). (b) Analysis of PDs obtained by MF-GCs (upper), ENDO-GCs (centre) and POR-GCs (lower) in the three different media. Values are means ± SD of at least six experiments in triplicate. PDs were calculated as described in the Methods Section. Data were analyzed by one-way ANOVA followed by Dunnett’s post hoc test. * *p* < 0.05, *** *p* < 0.001 comparison vs. MF group. Table S1. Sequence of primers used in the quantitative real-time PCR analysis.

## Abbreviations

The following abbreviations are used in this manuscript:

GCs	Granulosa Cells
FF	Follicular Fluid
FSH	Follicle stimulating hormone
E2	Estradiol
LH	Luteinizing Hormone
P4	Progesterones
MF	Male Factor
ENDO	Endometriosis
POR	Poor Ovarian Responder
ECM	Extracellular Matrix
FF-CM	Follicular Fluid added Culture Medium
FF-MF	Culture Medium with follicular fluid from MF patients
AMH	Anti-Müllerian Hormone
CORT	Cortisol
