# Supplementary material for: Progesterone and IL-6 Expression Are Modulated by Follicular Fluid in Granulosa Cell Cultures
Source: Biomolecules. 2025 Nov 23;15(12):1646. doi: 10.3390/biom15121646 (PMC12730343; doi:10.3390/biom15121646)
Supplement: Supplementary file 1 [file biomolecules-15-01646-s001.zip › biomolecules-3947590-supplementary.pdf]

Article

Progesterone and IL-6 Expression Are Modulated by Follicular Fluid in Granulosa Cell Cultures

Loris Marin [1,†], Chiara Sabbadin [2,†], Claudia Maria Radu [3], Paola Brun [4], Carolina Frison [4], Giuseppe Gullo [5], Decio Armanini [2,6], Luciana Bordin [1,*], Eugenio Ragazzi [,6], Guido Ambrosini [1] and Alessandra Andrisani [1]

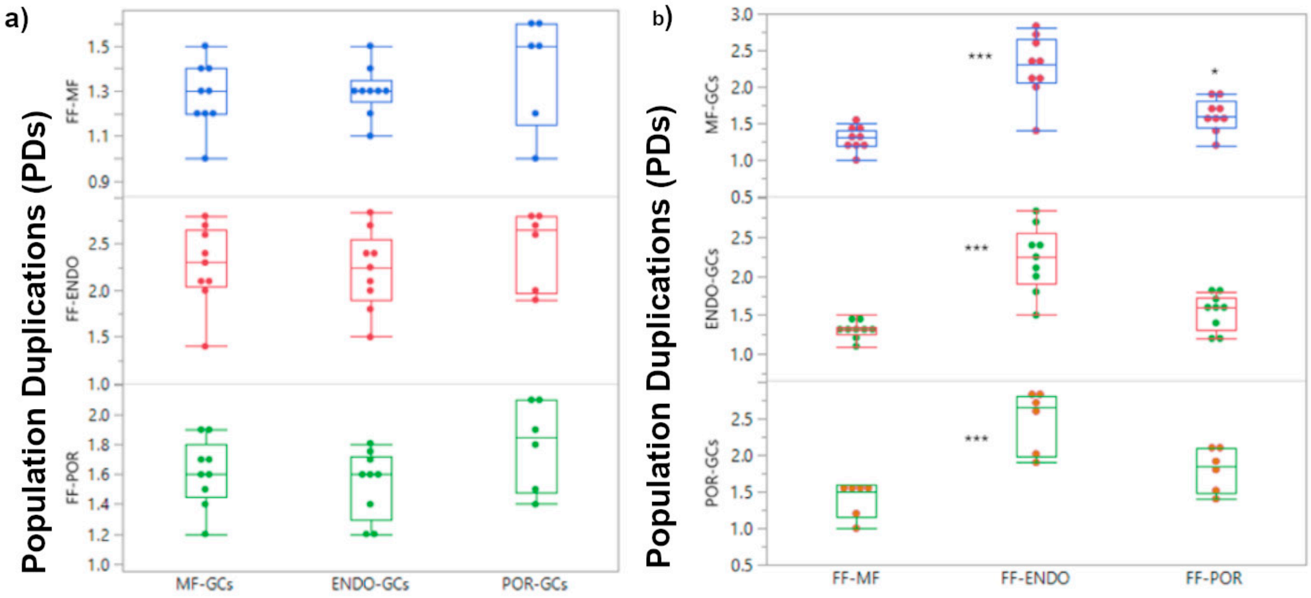

Figure S1. Population duplications (PDs) of the cells of each group (MF-GCs, ENDO-GCs and POR-GCs) in the culture medium supplemented with follicular fluid of MF (FF-MF), ENDO (FF-ENDO) and POR (FF-POR). a) Analysis of PDs obtained by each group of GCs in FF-MF (upper) FF-ENDO (centre) and FF-POR (lower). b) Analysis of PDs obtained by MF-GCs (upper), ENDO-GCs (centre) and POR-GCs (lower) in the three different media. Values are means ± SD of at least six experiments in triplicate. PDs were calculated as described in the Methods Section. Data were analyzed by one-way ANOVA followed by Dunnett's post hoc test. *$p < 0.05$, *** $p < 0.001$ comparison vs MF group.

Table S1. Sequence of primers used in the quantitative real-time PCR analysis.

Gene	Oligonucleotide (5′ – 3′)	
	forward	reverse
IL6	ACTCACCTCTTCAGAACGAATTG	CCATCTTTGGAAGGTTCAGGTTG
HSD3B1	GTCTTCGGTGTCACTCACAGAG	CTGGTGTAGATGAAGACTGGCAC
STAR	TCTCTACAGTGACCAGGAGC	GAACACCTTGCCCACATCTG
CCND1	TCTACACCGACACTCCATCCG	TCTGGCATTTTGGAGAGGAAGTG
B-ACTIN	CCAAGGCCAACCGCGAGAAGAT	AGGGTACATGGTGGTGCCGCCA

 https://doi.org/10.3390/biom15121646
